# Comparative analysis of p16 expression among African American and European American prostate cancer patients

**DOI:** 10.1002/pros.23833

**Published:** 2019-05-21

**Authors:** Myra Wong, Yaeli Bierman, Curtis Pettaway, Rick Kittles, Martha Mims, Jeffrey Jones, Michael Ittmann

**Affiliations:** ^1^ Department of Pathology and Immunology, Michael E. DeBakey VA Medical Center Baylor College of Medicine Houston Texas; ^2^ Department of Urology UT MD Anderson Cancer Center Houston Texas; ^3^ Department of Population Sciences, Division of Health Equities City of Hope Comprehensive Cancer Center Duarte California; ^4^ Department of Medicine Baylor College of Medicine Houston Texas; ^5^ Scott Department of Urology, Michael E. DeBakey VA Medical Center Baylor College of Medicine Houston Texas

**Keywords:** African American, p16, prostate cancer, TMPRSS2/ERG

## Abstract

**Background:**

Expression of p16 is increased in a number of malignancies, including prostate cancer (PCa). Recent studies in a European cohort showed that expression of p16 is correlated with expression of the TMPRSS2/ERG (T/E) fusion protein. The T/E fusion is significantly less common in PCas in African American (AA) men. Thus, it would be predicted that p16 expression should be less common in PCas in AA men. We, therefore, sought to compare the expression of p16 in benign prostate and PCas from AA and European American (EA) men.

**Methods:**

Immunohistochemistry for p16 and ERG was performed on tissue microarrays constructed from radical prostatectomies performed on AA and EA veterans. Staining was scored and the scores compared with demographic, clinical and pathological parameters. Percent of West African ancestry in the AA cohort was assessed using ancestry informative markers.

**Results:**

Contrary to our predictions, p16 expression was similar in the cancers in the AA and EA cohorts. Consistent with prior reports, expression of p16 was quite low in benign prostate tissues from EA patients but surprisingly was significantly higher in benign tissues from AA patients. Expression of p16 was significantly associated with a family history of PCa in AA men. In addition, p16 was associated with ERG expression in AA PCa.

**Conclusions:**

While overall expression of p16 is similar in PCas from the two racial groups, the expression of p16 in benign tissues from a subset of AA men and the stronger correlation with ERG expression implies that there are different mechanisms for p16 overexpression in PCas from the two racial groups.

## 1 INTRODUCTION

African American (AA) men have a significantly higher incidence of prostate cancer (PCa) compared with European American (EA) men and are twice as likely to die from PCa compared with EA men.^1^ Since AA men account for a significant fraction of all PCa related deaths in the United States, it is important to understand the increased mortality to optimize prevention and treatment strategies for this higher risk group of men. Data from autopsy studies demonstrate a higher incidence of high‐grade prostate intraepithelial neoplasia and PCa in AA men when compared with age‐adjusted EA men. AA patients exhibit greater tumor volumes in comparison to similarly staged EA patients^2^, ^3^ Several studies have evaluated differential expression of specific proteins including epidermal growth factor receptor, the androgen receptor (AR), cav‐1, and others among AA and EA cohorts.^4^, ^5^ A number of studies have compared gene expression in AA and EA PCa using large scale expression microarrays^6^, ^7^, ^8^, ^9^ including a study from our group.^10^ Other studies have focused on a smaller set of preselected genes.^11^, ^12^, ^13^ Of particular interest is the fact that the TMPRSS2/ERG (T/E) fusion gene is much less frequent in AA PCa.^12^, ^13^, ^14^, ^15^, ^16^, ^17^ These studies all indicate differential gene expression between AA and EA PCa and point to the fact that AA PCa is biologically distinct in many ways from EA PCa.

The p16 protein negatively regulates the cell cycle by inhibiting cyclin‐dependent kinase 4 (CDK4) and CDK6 interaction with cyclin D1. Increased p16 results in decreased retinoblastoma (Rb) protein phosphorylation preventing cell cycle progression from G1 to S.^18^, ^19^ p16 expression is lost in a variety of malignancies including bladder, pancreatic, colorectal, and lung carcinomas and melanomas.^18^, ^19^

Via feedback mechanisms, p16 protein is increased in a number of neoplasms in which the Rb protein is inactivated. In neoplasms arising from human papillomavirus (HPV) infection of the anogenital tract, HPV E7 inactivates Rb resulting in increased p16 expression which can be diagnostically useful. The p16 protein is also increased in a variety of non‐HPV‐related neoplasms including esophageal squamous cell^20^ and ovarian^21^ carcinomas and osteosarcomas.^22^ Presumably p16 has lost its tumor suppressive function in such cells via inactivation of Rb or other proteins involved in the Rb pathway, although the exact molecular mechanisms for increased p16 have not been defined in all cases.

Over the last 20 years, there have been a number of immunohistochemical studies of p16 expression in prostate and PCa.^23^, ^24^, ^25^, ^26^, ^27^, ^28^, ^29^ A variety of antibodies, staining protocols, and scoring systems have been used and studies range in size from several dozen to more than 9000 cancer cases. Smaller numbers of benign prostate tissues have also been analyzed. Overall these studies indicate that p16 protein is increased in PCa relative to benign tissues, with the exact percentage depending on the scoring system used and the specimen type. The impact on outcome is controversial, with some studies showing no impact while others show more aggressive disease in tumors expressing p16. These studies included primarily Caucasian patients, and p16 expression has not been examined in AA PCa and benign prostate in comparison to EA PCa. In this study, we examine the expression of p16 protein in PCa and benign prostate from AA and EA men.

## 2 MATERIALS AND METHODS

### 2.1 Tissue microarrays

Tissue microarrays (TMA) were constructed from radical prostatectomy tissues from AA and EA patients operated on at the Michael E. DeBakey VA Medical Center between 1995 and 2013. Patients provided written informed consent for the use of tissues under an Institutional Review Board approved the protocol. Areas of PCa and benign tissue were identified by pathological examination and 1 mm cores of cancer and matched benign tissues from each prostatectomy were used to construct TMAs. Demographic, clinical, and pathological information was abstracted from the patient's electronic medical record.

### 2.2 Immunohistochemistry

Immunohistochemistry (IHC) for p16 was carried out on a Leica BOND III autostainer using online heat treatment with ER1 antigen retrieval solution (citrate pH 6.0) for 20 minutes. Primary antibody incubation was carried out using E6H4 mouse monoclonal anti‐p16 antibody (Ventana, Tucson, AZ) for 15 minutes. Detection was carried out using a Bond Polymer Refine Detection Kit (Leica) for 16 minutes followed by chromogen for 5 minutes. Counterstain was hematoxylin. The positive control was a dysplastic lesion of the tonsillar epithelium. ERG IHC was carried out using the same basic protocol. Antigen retrieval was for 20 minutes using Bond Epitope Retrieval 2 (Leica, Grove, IL). The primary antibody was anti‐ERG mouse monoclonal antibody PM421AA (Biocare Medical, Pacheco, CA) which was incubated for 15 minutes. An ERG‐positive PCa was the external‐positive control and the internal‐positive control was endothelial cells in the prostate tissue sections. To avoid batch effects TMAs were stained in a single run for a given antibody. One of the AA TMAs consisted primarily of more recent cases (2011‐2013) compared with EA cases. This was done to increase the number of AA cases. Comparison of IHC results on this TMA compared with other AA TMAs did not show statistically different staining for p16 or ERG by analysis of variance on Ranks.

### 2.3 Analysis and scoring of IHC

A total of 171 cases from AA men and 189 from EA men were evaluable for p16 staining in benign and/or PCa tissues. ERG staining was evaluable in 175 cancers from AA men and 190 from EA men. Some cases were not evaluable due to loss of tissue or other artifacts. Luminal cells were scored for benign tissue and cancer epithelium scored for PCa. The intensity was scored from 0 to 3+, with 0 being absent staining, 1 weak, 2 moderate, and 3 strong. The extent of staining was also scored 0 to 3 based on the percentage of cells stained (0, <30%, 30%‐60%, or >60% as 0‐3, respectively). A multiplicative staining score was then calculated by multiplying the intensity time the extent to yield scores from 0 to 9. Staining was evaluated in both cytoplasm and nucleus independently for p16. For ERG staining nuclear staining in cancer cells was scored using the same system.

### 2.4 DNA extraction

Germline DNA was extracted from up to 25 mg of snap frozen seminal vesicle or benign prostate tissue using a DNeasy Blood and Tissue Kit (Qiagen, Germantown, MD) using the manufacturers protocol.

### 2.5 Genetic ancestry estimation

Agena Bioscience MassARRAY was used for genotyping 105 unlinked single nucleotide polymorphism ancestry informative markers (AIMs).^30^ iPLEX assays were designed utilizing the Assay Design software, allowing for single base extension (SBE) designs used for multiplexing. Multiplex assays were performed to amplify 5 to 10 ng of genomic DNA by polymerase chain reaction (PCR). Subsequently, a post‐PCR SBE reaction was performed for each multiplex reaction using concentrations of 0.625 μM for low mass primers and 1.25 µM for high mass primers. Reactions were dissolved with 16 µL of H_2_O and fragments purified with resin, spotted onto SpectroCHIP microarrays, and scanned by MALDI‐TOF mass spectrometry (Agena Bioscience, San diego, CA).

Genotypes from the enriched panel of 105 unlinked AIMs spanning 22 chromosomes was used to provide an estimation of genetic ancestry for each study subject. European Ancestry (EA), West African Ancestry (WAA), and Native American Ancestry (NAA) and was estimated from the genotype data using the Bayesian Markov Chain‐Monte Carlo (MCMC) method implemented in the program STRUCTURE version 2.1.^31^, ^32^

## 3 RESULTS

Tissues from a total of 191 EA and 180 AA tissues were used to construct the TMAs. All patients were diagnosed and treated at the Michael E. DeBakey VA Medical Center which is an equal access care setting in a uniform fashion. All radical prostatectomies were reviewed by a single pathologist (MI) as part of ongoing clinical care and thus pathological staging and grading are uniform. Patient demographics and clinicopathological characteristics are shown in Table 1. The AA patients were slightly younger (59.6 vs 61.2 years; *P* = 0.046, *t* test) and had significantly higher primary Gleason score (3.2 vs 3.0; *P* < 0.001). In addition, the total Gleason sum was higher in the AA patients (7.0 vs 6.8; *P* = 0.043). Of note, there was a highly significant increase in the proportion of AA patients with Grade group 3 (Gleason score 4 + 3 = 7) compared with EA patients (*P* < 0.001, *χ* ^2^). Preoperative prostate‐specific antigen (PSA) and final pathological stage were similar in the two racial groups, as was the clinical outcome. Biochemical recurrences and PCa specific deaths were not statistically significantly different between the two groups. Compared with the PIVOT prospective study of radical prostatectomy in a VA cohort that was approximately 30% AA,^33^ our PCa specific death is very similar at 8 years of follow‐up. This is in line with the overall low PCa specific mortality following radical prostatectomy in other cohorts. Our overall mortality was significantly lower than the PIVOT cohort (10% vs 20%). This may be due in part to the younger age of our patients (60 vs 67 years) as well as differences in patient selection for exclusion from radical prostatectomy based on comorbidities in the two cohorts.

**Table 1:** Clinicopathological characteristic of patients

	European American (n = 191)	African American (n = 180)	*P* value
Age	61.2 ± 0.39	59.6 ± 0.47	**0.046**
Body mass index	27.4 ± 0.34	27.6 ± 0.37	0.62
Family history of PCa	34 (17.8)	34 (18.9)	0.89
Preoperative PSA	7.9 ± 0.62	8.7 ± 0.82	0.34
Primary Gleason grade	3 ± 0.03	3.2 ± 0.03	**<0.001**
Gleason sum	6.8 ± 0.06	7.0 ± 0.05	**0.043**
Grade group 5	12 (6.3)	12 (6.7)	0.95
Grade group 4	1 (0.5)	2 (1.1)	0.96
Grade group 3	3 (3.1)	28 (15.6)	**<0.001**
Grade group 2	133 (68)	110 (61.1)	0.11
Grade group 1	42 (24)	28 (15.6)	0.15
Extracapsular extension	75 (39.1)	62 (33.7)	0.46
Seminal vesicle invasion	19 (9.9)	18 (9.8)	0.89
Positive surgical margin	56 (29.2)	54 (29.4)	0.7
Pelvic lymph node metastasis	6 (3.1)	2 (1.1)	0.32
Biochemical recurrence	56 (29.2)	36 (19.6)	0.054
Dead	24 (12.5)	14 (7.6)	0.178
Dead of prostate cancer	6 (3.1)	1 (0.6)	0.148
Total follow‐up (months)	96.3 ± 9.3	89.3 ± 3.9	0.48

*Note*: Values ± SEM or number with the percentage shown. *P* value by Mann Whitney or *χ* ^2^ test for African American vs European American is shown. Bold values are statistically significant using indicated statistical tests.

Assignment of the race was based on the patient's self‐reported race. We assessed the AIMs in the AA cases for which DNA was available after extraction from snap frozen benign tissue taken at the time of surgery. A total of 170 DNAs were analyzed. Mean West African ancestry was 77.2 ± 0.1 (SD)%, 6 ± 0.04% Native American, and 16.8 ± 0.1 European. The percentage of West African ancestry ranged from 50% to 93%. There was a 100% concordance between self‐identified race and the presence of significant West African heritage based on AIMs. These results are broadly concordant with results seen in other AA populations and confirm that the AA cases were from men with substantial West African genetic ancestry. We did not see any statistically significant correlations between clinical and pathological variables or results of the IHC studies with the percentage of West African ancestry. These results are summarized in Table S1. However, the cohort is relatively small and the clinical and pathological parameters do not show extensive variability since all patients were candidates for curative radical prostatectomy. In addition, more than 70% of AA men in our cohort fell between 70% and 90% West African lineage. Thus, the amount of variation was small in our cohort, limiting our ability to examine associations. Perhaps a much larger and/or more variable cohort might yield statistically significant associations.

### 3.1 Expression of p16 in AA and EA benign prostate and PCa tissues

**Figure 1:**
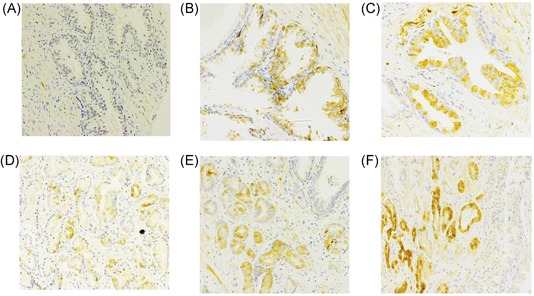
p16 immunohistochemistry in African American prostate cancer. A, Benign prostate, no staining. B, Benign prostate, moderate staining. C, Benign prostate, strong staining. D, Prostate cancer, weak staining. E, Prostate cancer, moderate staining. Normal prostate to upper right. F, Prostate cancer, strong staining. Normal prostate gland is on the right. Original magnification ×200 [Color figure can be viewed at wileyonlinelibrary.com]

The TMAs were stained by IHC with clinical grade p16 antibody using our established protocol. Staining was then scored. Both cytoplasmic and nuclear staining of p16 was assessed in benign prostate luminal epithelial cells and PCa cancer cells for all cases for which suitable tissue could be scored on the TMA. A total of 171 cases from AA men and 189 from EA men were evaluable in benign and/or PCa tissues. The intensity was scored from 0 to 3+, with 0 being absent staining, 1+ weak, 2+ moderate, and 3+ strong staining. The extent of staining was also scored 0 to 3 based on the percentage of cells stained (0, <30%, 30%‐60%, or >60% as 0‐3, respectively). A multiplicative staining score calculated by multiplying intensity times extent yielding scores from 0 to 9. Scores of 7 to 9 were considered strong staining, 4 to 6 as moderate staining, and 1 to 3 as weak staining. Examples of staining in benign prostate and PCa are shown in Figure 1. Both the intensity and extent of staining were quite variable in both groups and scores from 0 to 9 were seen across all groups. Cytoplasmic and nuclear staining scores were highly correlated within groups. Cytoplasmic and nuclear staining in benign tissues from AA and EA benign tissues had a correlation coefficient of *r* = 0.85 (*P* < 0.0001, Pearson Product Moment). Similar but slightly lower correlations were seen in cancer groups (*r* = 0.64 and 0.81 for AA and EA, respectively; Table 2). We also observed occasional staining of basal cells and spindle cells within the stroma, but these were not scored.

**Table 2:** Correlation between cytoplasmic and nuclear staining is benign prostate and prostate cancer cells in samples from African American and European American patients

	Benign		Cancer	
	Cytoplasmic	Nuclear	Cytoplasmic	Nuclear
*African American*				
Benign				
Cytoplasmic	1	**0.85; *P* < 0.0001**	0.15; *P* = 0.09	**0.38; *P* < 0.001**
Nuclear	X	1	0.11; *P* = 0.19	**0.49; *P* < 0.001**
Cancer				
Cytoplasmic	X	X	1	**0.64; *P* < 0.0001**
Nuclear	X	X	X	1
*European American*				
Benign				
Cytoplasmic	1	**0.85; *P* < 0.0001**	0.03; *P* = 0.69	0.02; *P* = 0.73
Nuclear	X	1	0.07; *P* = 0.38	0.03; *P* = 0.73
Cancer				
Cytoplasmic	X	X	1	**0.81; <0.0001**
Nuclear	X	X	X	1

*Note*: Correlation coefficients and *P* values for the Pearson Product Moment test for various comparisons are shown.

Bold values are significant based on Perason Product Moment test.

**Figure 2:**
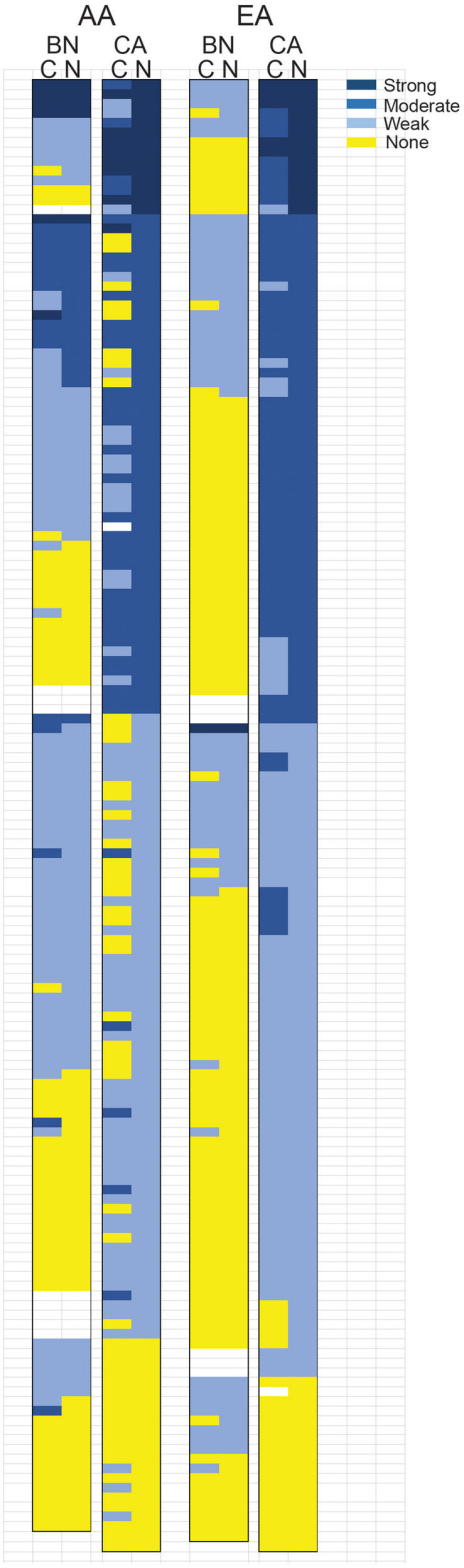
Heat map of p16 staining in African American and European American prostate cancer. Heat map with cases as individual rows. Staining intensity is indicated by the shade of blue as shown, with dark blue indicating strong staining (7‐9), medium blue moderate staining (4‐6), light blue weak staining (1‐3), and yellow, no staining. Cases within each racial group arranged by the intensity of cancer nuclear staining. AA, African American; BN, benign tissue; C, cytoplasmic staining; CA, cancer tissue; EA, European American; N: nuclear staining [Color figure can be viewed at wileyonlinelibrary.com]

**Figure 3:**
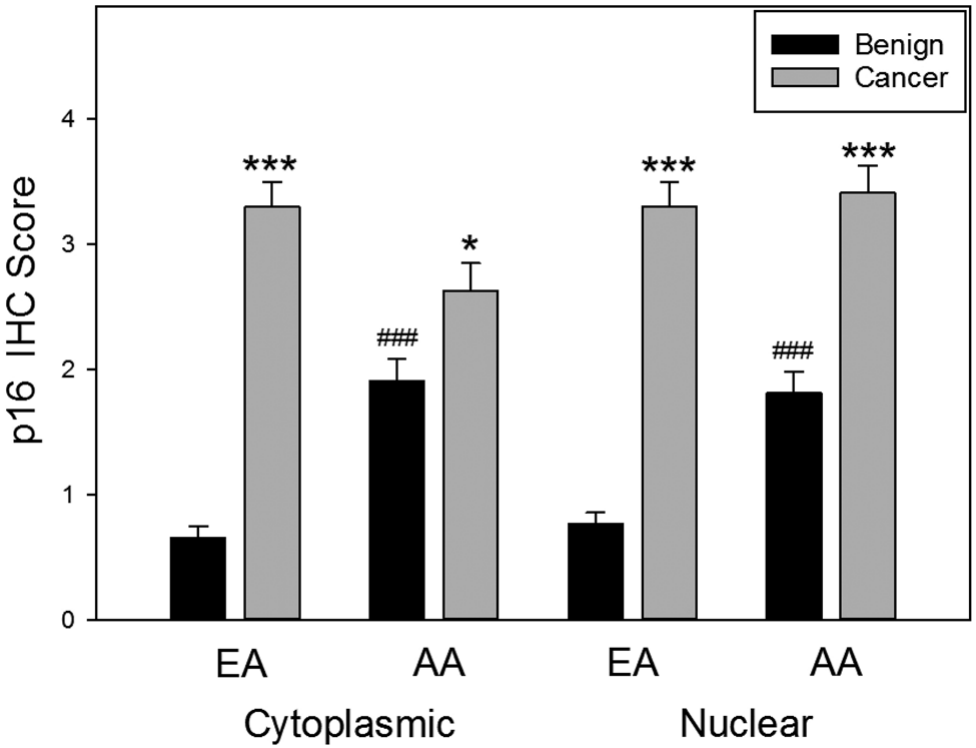
Cytoplasmic and nuclear staining scores in AA and EA prostate cancer. Mean ± SEM of p16 cytoplasmic and nuclear staining is shown. Asterisks indicate statistically significant differences between benign and cancer within each racial group by the cellular compartment. ***P < 0.001 and *P < 0.05; ^###^Mann Whitney test's statistically significant difference (*P* < 0.001, Mann Whitney) between AA and EA benign tissues in both cellular compartments. AA, African American; EA, European American; IHC, immunohistochemistry

Figure 2 shows a heat map of p16 scores in cytoplasm and nuclei for benign tissue and cancer tissue in AA and EA cohorts arranged by cancer nuclear staining scores. Nuclear staining scores were almost identical in AA and EA cancers with 9% showing strong staining (score 7‐9) and 35% showing moderate staining (score 4‐6) and the remaining cases showing weak (score 1‐3) or no staining. As shown in Figure 3, for EA PCa, p16 staining scores were significantly higher in both the cytoplasmic and nuclear compartments than the same compartment in benign tissue (*P* < 0.001, Mann Whitney). Cancer scores in AA cancer tissues were also significantly higher in the nucleus (*P* < 0.001, Mann Whitney) and cytoplasm (*P* < 0.05, Mann Whitney) compared with benign tissues in tissues from AA men.

Consistent with prior reports, the EA benign tissues were only weakly or not stained for p16 protein except for one case which showed strong staining (Figure 2). Surprisingly, the AA benign tissues showed strong nuclear staining in 4% of cases and moderate staining in 13% of cases (Figure 2). Consistent with the observations described above, the AA benign tissue showed significantly stronger staining in both the nucleus and cytoplasm compared with EA benign tissues (*P* < 0.001, Mann Whitney; Figure 3). Of note, cytoplasmic and nuclear staining in benign tissues were strongly correlated with nuclear staining in cancer tissues in AA tissues (*r* = 0.38 and 0.49, respectively, both *P* < 0.001; Table 2). Examination of the heat map of staining scores in Figure 2 shows that 22 of 27 AA benign cases with strong or moderate staining in the cytoplasm and/or nuclei of benign cells had strong or moderate p16 staining in the cancer nuclei, consistent with the observed correlation described above. However, in tissues from EA patients, no correlation was seen between staining in cytoplasmic or nuclear staining in benign tissues and nuclear (or cytoplasmic) staining in cancer cells. Overall these results indicate that p16 staining is significantly higher in AA benign tissues and this is associated with p16 nuclear expression in PCa tissues in these patients in many cases.

### 3.2 Expression of p16 in AA benign prostate and PCa tissues is associated with a family history of PCa

**Figure 4:**
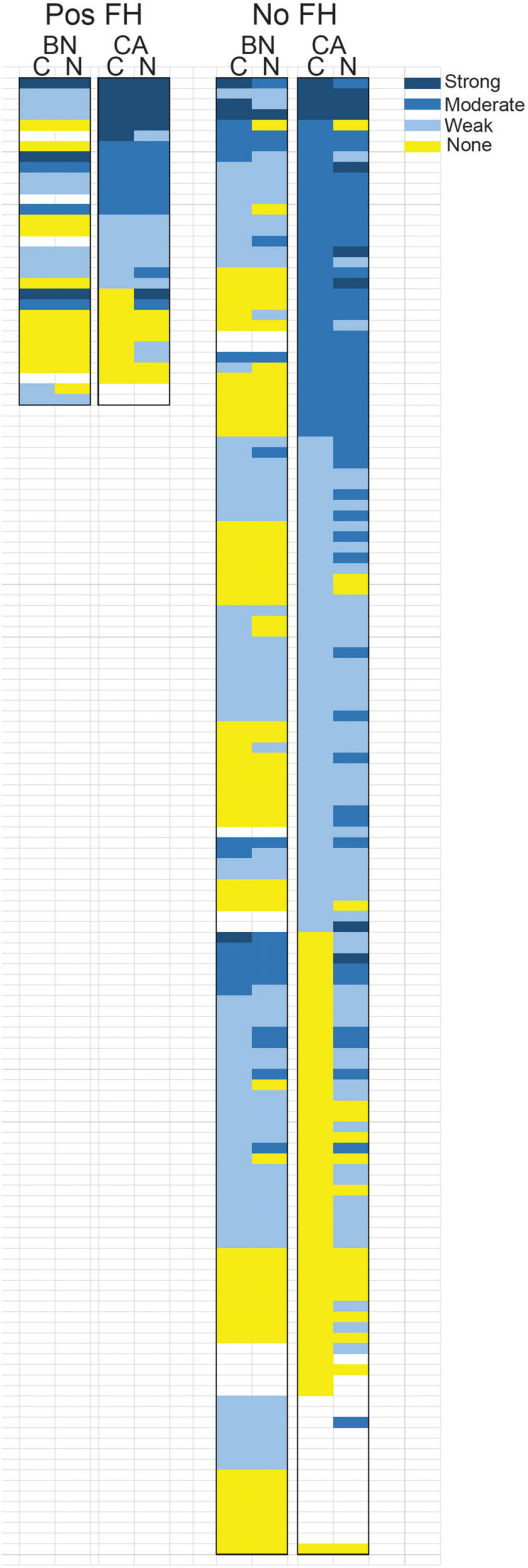
Heat map of p16 staining in African American with or without a family history of prostate cancer. Heat map with cases as individual rows. Staining intensity is indicated by the shade of blue as shown, with dark blue indicating strong staining (7‐9), medium blue moderate staining (4‐6), light blue weak staining (1‐3) and yellow, no staining. Cases within each family history group are arranged by intensity of cancer cytoplasmic staining. BN, benign tissue; C, cytoplasmic staining; CA, cancer tissue; N, nuclear staining; No FH, no family history of prostate cancer; Pos FH, positive family history of prostate cancer [Color figure can be viewed at wileyonlinelibrary.com]

We next examined the correlation of p16 with various clinical and pathological parameters in both AA and EA patients. We did not observe any association of p16 staining in nuclei or cytoplasm of benign or cancer tissues with age, body mass index, preoperative PSA, Gleason score, extracapsular extension, seminal vesicle invasion, pelvic lymph node metastasis, biochemical recurrence, or death from PCa or other causes (Table S2). However, we did find a significant correlation of family history of PCa in AA patients, with p16 expression in benign tissues and cytoplasmic staining in cancer (Table 3A). Nuclear staining in cancer trended toward a positive correlation (*P* = 0.06). This is shown in the heatmaps in Figure 4 comparing p16 staining in AA benign and cancer tissues in patients with or without a family history of PCa. There is clearly a much higher percentage of cases with moderate to strong staining in prostates from men with a family history of PCa, particularly in cancer tissues. Based on this observation, we analyzed the proportion of cases with strong p16 staining in men with or without a family history of PCa. There was a significant association of family history of PCa with strong nuclear staining in benign tissues (Table 3B).

**Table 3:** Association of p16 staining with a family history of prostate cancer

		Benign		Cancer	
		Cytoplasmic	Nuclear	Cytoplasmic	Nuclear
A.	African American	**0.173; *P* = 0.031**	**0.169; *P* = 0.035**	**0.207; *P* < 0.01**	0.152; *P* = 0.06
	European American	(0.03); *P* = 0.617	0.01; *P* = 0.519	(0.06); *P* = 0.47	(0.08); *P* = 0.32
B.	African American	*P* = 0.184	***P* = 0.015**	***P* = 0.002**	***P* = 0.042**
	European American	NA	NA	*P* = 0.983	*P* = 0.606

*Note*: (A) Correlation of p16 score and family history of prostate cancer; correlation coefficients and *P* values are shown for Pearson Product Moment test. (B) *P* value of *χ* ^2^ tests comparing fraction of tissues with strong staining in men with or without a family history of prostate cancer.

Bold values are statistically significant using indicated statistical tests.

### 3.3 Increased p16 messenger RNA in PCa

**Figure 5:**
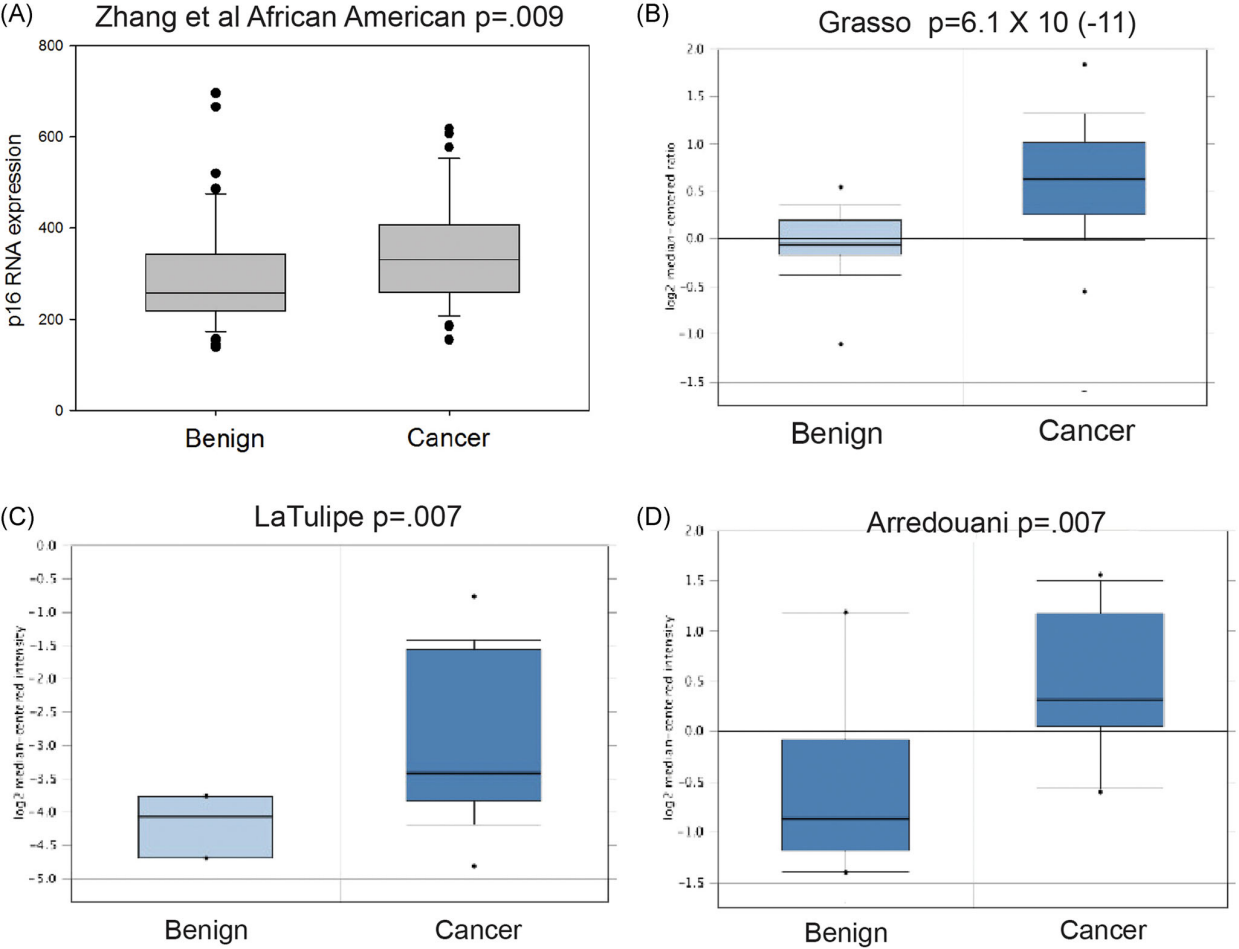
CDKN2A (p16) mRNA in benign prostate and prostate cancer tissues. A, Bar and whisker plot of CDKN2A (p16) mRNA expression from expression microarray data of 48 African American benign prostate tissues and prostate cancers; *P* = 0.009, *t* test. B‐D, Bar and whisker plots of log_2_ transformed expression data for prostate and prostate cancer from Oncomine. The *P* value for differences between benign and cancer is shown as is the name of the database [Color figure can be viewed at wileyonlinelibrary.com]

The IHC studies above show that p16 protein is increased in both AA and EA PCa. This could be due to increased p16 messenger RNA (mRNA) or potentially to posttranscriptional factors such as increased translation**,** increased protein stability, and so forth. We, therefore, examined our previously published expression microarray data that examined gene expression in 48 AA PCa and benign tissues^10^ for CDKN2A (p16 gene) expression. Expression was 1.2‐fold higher in PCa (*P* = 0.009, *t* test) as shown in Figure 5A. We then examined CDKN2A expression in PCa datasets in Oncomine. Of 14 data sets with mRNA data, all from predominantly EA cohorts, 8 of 14 showed a statistically significant increase in CDKN2A (examples shown in Figure 5B‐D) and only 1 of 14 showed a statistically significant decrease. Thus, the increased p16 protein expression in both AA and EA patients is due, at least in part, to increased p16 mRNA levels in the PCa tissues.

### 3.4 Expression of ERG in AA and EA PCa tissues

**Figure 6:**
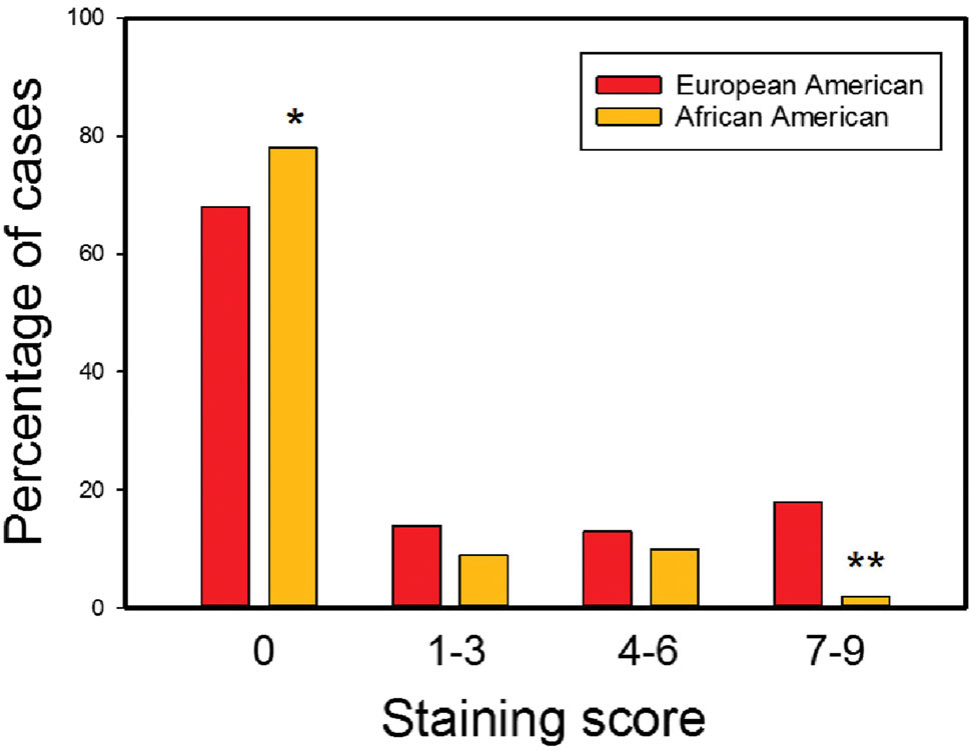
ERG expression in AA and EA prostate cancer. Percentage of prostate cancers with no staining (0), weak staining (1‐3), moderate staining (4‐6), or strong staining (7‐9) in EA and AA patients. *P < 0.05; **P < 0.01, *χ* ^2^. AA, African American; EA, European American [Color figure can be viewed at wileyonlinelibrary.com]

The same set of TMAs was then stained with an anti‐ERG antibody to evaluate the expression of the T/E fusion gene using IHC. We and others have shown that there is variability in the intensity and extent of ERG staining in PCa.^34^ Therefore, the intensity was scored using the multiplicative staining index as described for p16 except only nuclear staining in cancers was scored. Scores of 7 to 9 were considered strong staining, 4 to 6 as moderate staining and 1 to 3 as weak staining. Data for both races is shown in Figure 6. As expected, AA PCa had a significantly lower proportion of ERG‐positive cases (38 of 175 evaluable AA cases vs 60 of 190 evaluable EA cases, *P* = 0.024, the *χ* ^2^ test). Unexpectedly, there was a significant difference in the proportion of cases with high staining intensity (≥7), with AA PCa having only a few strongly staining cases (4 of 175 vs 18 of 190, *P* < 0.01, *χ* ^2^). Prior studies have focused on expression, not differences in ERG expression levels in AA and EA PCa. The reason for the observed racial difference is unclear. The T/E fusion protein expression level is controlled at both the transcriptional and posttranscriptional level, but a detailed understanding of control in human PCa tissues is still lacking. There was also a significantly higher proportion of cases with no staining as expected (*P* < 0.05, *χ* ^2^).

The association of the variables in Table 1 with ERG expression was examined for both racial groups. ERG staining in EA PCa was strongly associated with extracapsular extension (34 of 60 ERG positive with extracapsular extension vs 41 of 130 ERG negative; *P* < 0.001, *χ* ^2^). There was also an association with higher Gleason score, with ERG‐positive cases have a slightly higher Gleason score (6.97 vs 6.83; *P* = 0.031, Mann Whitney). This was due primarily to a significantly lower fraction of Grade group 1 case in the ERG positive cases (7 of 60 vs 34 of 130; *P* = 0.039, *χ* ^2^). These pathological parameters are associated with more aggressive disease, although we did not see any association of ERG expression with clinical parameters in EA men such as PSA recurrence. The impact of ERG expression on disease aggressiveness in PCa is controversial. The exact reason for this discordance is unclear and may reflect differences in case selection, patient populations, and/or outcome measures evaluated. A recent meta‐analysis by Song and Chen^35^ concluded that the T/E fusion gene was associated with T3‐4 pathological stage but not with clinical outcome, similar to our results. No significant association of variables in Table 1 with ERG status in AA PCa was seen. The percent West African ancestry was also not associated with ERG status (Table S1).

### 3.5 Expression of p16 is correlated with ERG status in AA PCa

Finally, we examined the correlation of p16 cytoplasmic and nuclear staining in cancer cells with ERG expression in AA PCa. Interestingly, there was a significant correlation of p16 staining with ERG staining. Spearman Rank Order correlation for p16 cytoplasmic staining with ERG staining was *r* = 0.484 with *P* < 0.001 and for p16 nuclear staining was *r* = 0.259 with *P* < 0.01. The mean p16 nuclear staining in ERG‐positive cases was significantly higher than in ERG‐negative cases (4.69 vs 2.94; *P* < 0.001, Mann Whitney) as was cytoplasmic staining (4.91 vs 2.3; *P* < 0.001). No significant correlation was seen in EA PCa and there was no significant difference in the mean p16 nuclear or cytoplasmic staining in ERG positive and ERG negative cases from EA men.

## 4 DISCUSSION

There have been multiple studies of p16 protein expression in EA and European prostate and PCa cohorts by IHC.^23^, ^24^, ^25^, ^26^, ^27^, ^28^, ^29^ To our knowledge ours is the only study of p16 protein expression in AA prostate tissue and PCa. In the EA and European cohorts, increased p16 protein in PCa compared with benign prostate has been a consistent finding. The largest study was of a German cohort of 9627 PCas on TMAs.^23^ Of note, this group found a strong association between the presence of the T/E fusion and associated ERG expression and p16 expression, although the exact mechanism of this association is unknown. AA PCa has a significantly lower incidence of the T/E fusion compared with EA PCa^12^, ^13^, ^14^, ^15^, ^16^, ^17^ and we have confirmed this in the current study. Thus, it would be predicted that AA PCa should have a lower p16 expression than EA PCa. Contrary to our initial hypothesis, that p16 should be lower in AA PCa, we have found that overall p16 expression in cancer tissues is similar in the two racial groups. However, there are distinct differences the associations with p16 expression in AA and EA PCa.

While ERG expression is indeed lower in AA PCa there is an increased proportion of p16 expressing cases among the ERG expressing AA PCas when compared with EA PCa. This increased propensity to coexpress p16 and ERG‐positive PCa in AA patients in part compensate for the lower fraction of ERG‐positive cancers to make the EA and AA PCa more equal in p16 expression. Why the two racial groups differ in the strength of this association is not known.

Our data also shows that p16 expression is increased in benign prostate epithelial cells in AA men with PCa compared to benign prostate tissues in EA men and that this increased p16 in benign tissues is correlated with p16 expression in PCa. Thus, for at least a subset of AA PCa, there is increased p16 expression in benign tissues, perhaps due to inactivation of the Rb pathway in the benign cells that subsequently give rise to cancers with the same alterations. Genetic alterations can occur in benign epithelium in patients without any histological alterations in the benign cells as a field effect.^36^ Furthermore, our data shows that in a subset of patients with increased p16 expression in benign epithelium and their associated cancers there is an association with a family history of PCa. Thus, we hypothesize that AA men are more prone to developing loss of the Rb pathway activity with subsequent increased p16 and, in some cases, this is due to hereditary factors. We have shown previously that AA PCa has significantly higher rates of loss of 13q13.1 to 13q14.3,^37^ which contains the Rb locus.

An alternative hypothesis is suggested by the finding of Burdelski et al^23^ of a strong positive correlation of AR expression and p16 staining. Several groups have reported increased AR protein^5^ or mRNA^13^ in AA PCa. Thus, AR may be directly or indirectly driving higher p16 expression in AA benign and malignant prostate and in some cases, AR activity may be associated with germline variation in AA men. Presumably, the inhibitory effects of p16 are abrogated in this context. Further studies are needed to determine the mechanistic basis of the correlations observed in this study.

We did not see any impact of p16 expression on outcome following radical prostatectomy in AA or EA cohorts. In this disease setting, several groups have found an association of p16 expression with biochemical recurrence and/or PCa specific survival^23^, ^25^, ^29^ while other groups have not observed this association.^25^, ^26^ As discussed by Remo et al,^25^ this may be due in part to factors such as the IHC protocol, the scoring system, group choice for analysis and patient selection. In addition, the size of the cohort and the length of follow‐up may impact whether such associations are seen, particularly if they are relatively weak. Examination of the very large cohort of Burdelski et al^23^ suggests that p16 expression has an impact on the outcome, but mainly in the ERG negative cases within this European cohort. We did not see this association (data not shown) but our cohort is much smaller.

## 5 CONCLUSIONS

While p16 expression in cancer tissues is similar in AA and EA PCa tissues, p16 expression is more strongly associated with ERG expression in AA men and is associated with increased p16 expression in benign tissues. These findings suggest significant differences in the Rb/p16 pathway in AA and EA that warrant further investigation.

## CONFLICT OF INTERESTS

The authors declare that there is no conflict of interests.

## Supporting information

Supporting information

Supporting information
