# Supplementary material for: Comparative analysis of p16 expression among African American and European American prostate cancer patients
Source: Prostate. 2019 May 21;79(11):1274–83. doi: 10.1002/pros.23833 (PMC6617792; doi:10.1002/pros.23833)
Supplement: Supplementary file 1 — Supporting information [file PROS-79-1274-s001.docx]

|  |  |  |
| --- | --- | --- |
|  |  |  |
|  | **Correlation** | **p-value** |
| European ancestry (%) | **-0.929** | **<.000001** |
| Age | -0.099 | 0.2 |
| Body Mass Index | 0.028 | 0.71 |
| Family History of Prostate Cancer | -0.102 | 0.103 |
| Pre-operative PSA | 0.009 | 0.909 |
| Primary Gleason Grade | -0.021 | 0.784 |
| Gleason Sum | -0.11 | 0.168 |
| Extracapsular Extension | -0.065 | 0.403 |
| Seminal Vesicle Invasion | 0.033 | 0.671 |
| Positive Surgical Margin | -0.002 | 0.977 |
| Pelvic Lymph Node Metastasis | 0.065 | 0.403 |
| Dead | -0.011 | 0.883 |
| Dead of Prostate Cancer | -0.04 | 0.609 |
| p16 Benign (nuclear) | -0.08 | 0.341 |
| p16 Cancer(nuclear) | 0.008 | 0.921 |
| ERG Cancer | -0.007 | 0.933 |

**Supplementary Table 1.**

Correlation of demographic, clinical and pathological parameters and immunohistochemistry scores with percent West African ancestry using Pearson Product Moment test. Correlation coefficient and p-value are shown. European ancestry was strongly negatively correlated with West African ancestry; all other p-values were not significant.
