# Supplementary material for: Comparative analysis of p16 expression among African American and European American prostate cancer patients
Source: Prostate. 2019 May 21;79(11):1274–83. doi: 10.1002/pros.23833 (PMC6617792; doi:10.1002/pros.23833)
Supplement: Supplementary file 2 — Supporting information [file PROS-79-1274-s002.docx]

**Supplementary Table 2.**

Correlation of demographic, clinical and pathological parameters with p16 nuclear immunohistochemistry scores using Pearson Product Moment test in European American and African American men. Correlation coefficient and p-value are shown.
